# Adaptive migration promotes food web persistence

**DOI:** 10.1038/s41598-019-49143-8

**Published:** 2019-09-02

**Authors:** A. Mougi

**Affiliations:** Institute of Agricultural and Life Sciences, Academic Assembly, Shimane University, 1060 Nishikawatsu-cho, Matsue, 690-8504 Japan

**Keywords:** Food webs, Ecological modelling

## Abstract

Interactions between diverse species that coexist in nature are of utmost interest in the field of ecology. Recent theoretical studies have shown that spatiality plays a key role in maintaining complex systems with multiple differing species. In these models, however, organisms move among habitats randomly, implying that some organisms migrate from areas of higher fitness to areas of lower fitness in a maladaptive way. Herein, a meta-community model of a food web shows that adaptive movements by organisms can play key roles in maintaining large ecological communities. Without adaptive dispersal, species are not likely to persist across habitats, particularly when systems have few habitats where local food webs are strongly coupled by high migration rates. However, adaptive dispersers can improve such low persistence greatly. By abandoning unfavourable habitats for favourable habitats, dispersers prevent regional extinction at the price of local extinction and increase their total numbers further. Hence, the inherent stabilising effect of spatiality may be larger than that expected from theoretical random movement models.

## Introduction

Food webs are formed between collections of species that are linked by trophic interactions and are key units of biodiversity^1^. Previous ecological studies have explored the dynamics of food webs and their stability to investigate the mechanisms by which biodiversity is maintained^2–5^. Food web dynamics are driven by intrinsic birth–death processes influenced by species interactions^6–8^. Alternatively, food web dynamics can be viewed in terms of networks of local food webs that are connected by species movements^9,10^. Movements that are directed at procuring resources and avoiding natural predators are also inherent drivers of food web dynamics^11–13^. Thus, the dynamics of whole food webs can be driven by species interactions within local food webs and by movements between these^14–17^. Yet, assessments of complex spatial dynamics of very large food webs are a challenge for community ecologists^18,19^.

Recent theoretical studies have addressed this complex problem in part^20–26^. Most of these studies suggest positive effects of spatiality on food web maintenance^23,24^. However, these studies are based on strong assumptions of random species migration. In random migration models, spatial coexistence depends on maladaptive decisions of some individuals, with net movements from areas of higher fitness to those of lower fitness^10^. On the contrary, organisms often change their habitats to avoid high predation risk^11^ or to seek key resources^12,13^. Hence, these adaptive movements represent mechanisms by which population dynamics are influenced by resources and consumers, even without death due to predation and/or birth due to consumptions^27–30^. A number of theoretical studies consider adaptive habitat choices as key drivers of prey–predator dynamics^15,31,32^, but the associated analyses are biased to simple food web modules comprising only a few species^10^, or some studies using complex food web with diverse species focused on the effects of non-random migration to community structures such as spatial distribution^33–35^. Hence, the impacts of non-random adaptive movements on multi-species food web dynamics and its persistence are poorly understood.

In the present study using a food web model with adaptive movements, it was demonstrated that compared with random non-adaptive movements, adaptive movements had greater positive effects on community persistence. To this end, a food web comprising *N* species in which any pair of species are connected to each other with probability *C* was represented using a cascade model^36^ as a simple interaction network structure. In this model, for each pair of species *i*, *j* = 1, …, *N* and *i* < *j*, species *i* never consumes species *j* but species *j* may consume species *i*, and populations of each species can move freely between habitats. In these analyses, habitats are assumed to be heterogeneous and no within-species parameter correlations were considered among habitats^24^. The heterogeneity between habitats is assumed by the differences in growth rates (a demographic parameter) and consumption rates (interaction strength) (Methods). A complete graph was used to model the habitat network structure. The strength of species migration between local food web areas is given as *M*. Adaptive movements were also modelled so that movements between resource areas are motivated by differences in fitness or per-capita growth rates (see Methods). The novel fitness sensitivity parameter *θ* was introduced to interpret adaptive ability and to incorporate habitat quality, and is hereafter referred to as adaptive ability. Accordingly, more adaptive dispersers with larger *θ* values (>0) can move correctly depending on fitness differences between habitat areas. If no information pertaining to habitat qualities is available, *θ* = 0. Proportions of adaptive dispersers (*θ* > 0) and non-adaptive dispersers (*θ* = 0) within a community are controlled by *p*_A_, which represents the proportion of adaptive dispersers within a community. Community persistence was calculated as the probability that all species persist for a given time and was used as an index of food web stability^37^. When total population sizes become low, the species is defined as being extinct (Methods). Because the goal of these computations was to identify roles of adaptive movements in community persistence, the proportion of adaptive dispersers within a community *p*_A_, the spatial coupling strength *M*, and the adaptive ability *θ* were systematically controlled in the model.

## Results

**Figure 1:**
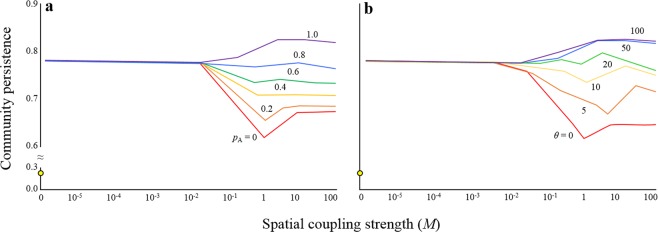
Relationships between spatial coupling strengths (*M*) and persistence; (**a**) effects of adaptive dispersers (*p*_A_) in which adaptive ability *θ* = 50; (**b**) effects of adaptive ability *θ* when *p*_A_ = 1.0. Yellow dots represent the community persistence in a special case where *M* = 0. Persistence was calculated along values of *M*, range of which is 0 and from 10^−5^ to 10^2^ (total 65 points). *N* = 20, *C* = 0.5 and *H*_*N*_ = 2.

Initially, the simplest scenario of two local food webs was considered. In an extreme case where the food web has no adaptive dispersers (*p*_A_ = 0 or *θ* = 0), local food webs are isolated (*M* = 0) and species have low persistence (Fig. 1). Upon connection of local food webs by random dispersers (*M* > 0), high-level persistence is immediately consequent. But further increases in spatial coupling strengths dramatically decrease this persistence, potentially because strong coupling transforms the behaviour of the meta-food web into that of a single food web. This unimodal pattern of persistence was also found in a random food web (see Supplementary Fig. S1).

When local food webs are loosely coupled by low migration rates, introduced adaptive dispersers have limited effects on persistence (Fig. 1a), but when spatial coupling strength exceeds a threshold, adaptive dispersers can contribute remarkably to community persistence. Moreover, in the presence of adaptive dispersers, loss of persistence is recovered in strongly coupled food webs. As proportions of adaptive dispersers within the community increase, food webs become increasingly persistent and stabilisation effects reach a level at which community persistence is impervious to spatial coupling strength (*p*_A_ = 0.8). However, further increases in proportions of adaptive dispersers within food webs can reverse the otherwise negative effects of coupling strength on community persistence. The resulting positive effect of coupling strength on persistence is likely to occur particularly when the adaptive abilities of dispersers are high (Fig. 1b) and the food web network is cascade (non-random) (see Supplementary Fig. S1).

**Figure 2:**
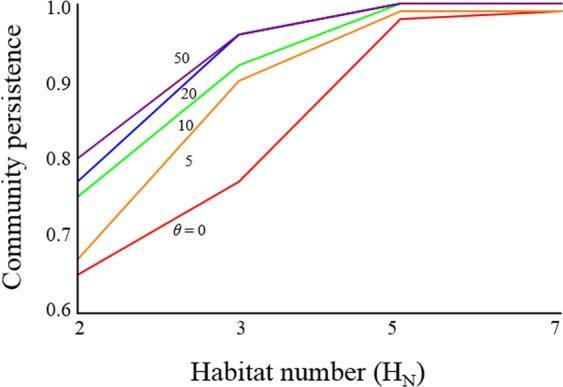
Effects of habitat numbers (*H*_*N*_) on persistence; colours represent different levels of adaptive ability (*θ*); *N* = 20, *C* = 0.5, *M* = 1.0 and *p*_A_ = 1.0.

More complex cases with more than three local food webs were considered in further computations. These showed three major effects of habitat number (Fig. 2). First, persistence increases with increasing numbers of local food webs, irrespective of whether movements are random (*θ* = 0) or adaptive (*θ* > 0). Second, persistence increases with adaptive ability. Third, the positive effect of adaptive dispersers on persistence is dependent on the number of distinct habitats. In food webs with fewer habitats, persistence can be greatly recovered by adaptive dispersers. The resulting increases in persistence of food webs with greater numbers of habitats and increasing adaptive abilities are limited, however, because persistence is high without adaptation.

Adaptive movements can increase community persistence by preventing regional extinction across all habitats. But when all species are adaptive dispersers and migrate from habitats of lower fitness to those of higher fitness, local populations with lower fitness become extinct, despite the simultaneously prevention of regional extinction. Hence, local extinctions are more likely to occur when adaptive ability is high (see Supplementary Fig. S2). In contrast, local extinctions are not likely to occur in the presence of multiple habitats, potentially because large variations of habitat provide multiple places at which dispersers’ fitness is increased. Furthermore, by migrating to avoid greater mortality and/or lower reproduction, adaptive dispersers allow overall abundances of each species to increase, particularly in food webs with many habitats (see Supplementary Fig. S3). These observations suggest that adaptive movements support food webs by both reducing regional extinction and increasing population abundances.

## Discussion

The present study shows that adaptive migration contributes more to the maintenance of community than random non-adaptive movements. In particular, the positive effects of adaptive dispersers on persistence tend to be present in systems with strong spatial coupling and high migration activities and in spatially simple systems with few habitats. Adaptive movements prevent regional extinction at the cost of local extinction by avoiding habitats in which greater mortality and/or lower reproduction occur, causing an increase in overall abundance and greater regional diversity.

Although adaptive movements can reduce local diversity, they increase regional diversity, and thus play a key role in maintaining regional food webs. Specifically, local extinction due to adaptive migration can lead to small sizes of local communities, thus making higher persistent local food webs by virtue of the greater persistence of smaller systems, as predicted by May^38^. In contrast, because random or non-adaptive movements do not reduce local population diversities, realised local communities remain large, resulting in decrease in food web persistence. This instability can be mitigated by increased numbers of habitats, potentially due to increased numbers of areas with resources.

Whether adaptive movements have stronger persistent power than random or non-adaptive movements depends on spatial coupling strengths and habitat numbers. Adaptive movements contribute to community persistence, particularly when food webs are strongly integrated by high migration rates. Hence, the effects of adaptive dispersers on persistence are dependent on spatial scale. Adaptive movements may also be more important for community dynamics at small spatial scales, where organisms come and go from focal habitat areas with ease. This argument would be held when we appropriately select the spatial scale in such a way that beta diversity does not change. In contrast, if we select such narrow regions that extremely decrease the species richness and/or habitat heterogeneity, it should reduce or lose the effects of adaptive dispersers. These suggest that adaptive dispersers play a key role in community persistence in an intermediate spatial scale. The relative contributions of adaptive and random movements to community persistence, however, approach unity in food webs with multiple local habitat foci. Therefore, I suggest that the effects of adaptive movements on community persistence also depend on habitat complexity. In ecosystems with relative environmental simplicity, such as those at high latitudes^39^, adaptation may greatly contribute to community persistence^40,41^. In contrast, in ecosystems with environmental mosaicity, such as those at low latitudes^39^, high adaptive ability may contribute little to community persistence. In relatively simple environments, organisms need to correctly assess habitat qualities, because areas with highly favourable qualities are very limited. In contrast, complex environments, selection pressure for the ability to assess habitat qualities may be weak due to the ease of movement between favourable areas and the inability to assess multiple areas^42–44^. Hence, dispersal may be more adaptive at high latitudes than at low latitudes. These hypotheses will be tested in comparisons of adaptive dispersal abilities of community members between low and high latitudes^45^.

As the first step toward understanding the role of adaptive migration in the persistence of meta-food web, the present model makes a simplifying assumption, perfect graph of habitat network structure. In this extreme, adaptive dispersers can choose more profitable places from multiple habitats, allowing adaptive migration to effectively work. Hence, if the connectivity between habitats is low, the positive role of adaptive dispersers for community persistence would weaken. Considerations of more realistic network topology in various natural ecosystems into the model will be necessary to further understand the roles of adaptive migration in community dynamics.

The present study has important implications for biodiversity conservation. Habitat destruction is known to decrease community stability^23,24,46^. However, the associated impacts may depend on whether community members can adaptively move between habitats. That is, destabilising effects of habitat destruction should be stronger when adaptive dispersal is limited. Further studies are warranted to determine dispersal modes of community members and to predict the corresponding dynamic community responses to habitat destruction.

## Methods

I considered a food web in which pairs of species *i* and *j* (*i*, *j* = 1, …, *N*) are connected by a trophic interaction with probability *C*. The cascade model was used as a simple interaction network structure. In this model, for each pair of species *i*, *j* = 1, …, *N* with *i* < *j*, species *i* never consumes species *j* and species *j* may consume species *i*. The maximum link number *L*_max_ is calculated as *N*(*N –* 1)/2 and the spatial food web model is defined using the following ordinary differential equation:

1 $$\frac{d{X}_{il}}{dt}={X}_{il}\{{r}_{il}-{s}_{il}{X}_{il}+\mathop{\sum }\limits_{j=1}^{N}{a}_{ijl}{X}_{jl}\}+M(-\mathop{\sum }\limits_{k=1(l\ne k)}^{{H}_{N}}{{m}_{i}f}_{ilk}{X}_{il}+\mathop{\sum }\limits_{k=1(k\ne l)}^{{H}_{N}}{m}_{i}(1-{f}_{ilk}){X}_{ik}),$$

where *X*_*il*_ (*l* = 1…*H*_*N*_) (*H*_*N*_ is the number of patches) is the abundance of species *i* in habitat *l*, *r*_*il*_ is the intrinsic rate of change in species *i* in habitat *l*, *s*_*il*_ represents the density-dependent self-regulation of species *i* in habitat *l*, and *a*_*ijl*_ is the interaction coefficient between species *i* and species *j* in habitat *l*. Interaction coefficients are defined as *a*_*ijl*_ = *e*_*ijl*_*α*_*ijl*_ and *a*_*jil*_ = − *α*_*ijl*_, where *α*_*ijl*_ is the consumption rate and *e*_*ijl*_ (<1) denotes the conversion efficiency. In these equations, migration rates are the product of the scaling parameter for spatial coupling strength *M*, and the species-habitat specific emigration rate *m*_*i*_*f*_*ilk*_ and the immigration rate is expressed as *m*_*i*_(1 - *f*_*ilk*_), where *k* = 1 … *H*_*N*_ but *k* ≠ *l*. *m*_*i*_ is the species-specific maximum migration rate. *f*_*ilk*_ is calculated as follows^47^:

2 $${f}_{ilk}=\frac{1}{1+{e}^{\theta ({W}_{il}-{W}_{ik})}},$$

where *W*_*ij*_ (*j* = *l or k*) is the fitness of each population within a habitat, defined as *W*_*ij*_ = *r*_*il*_ − *s*_*il*_*X*_*il*_ + Σ_*j*_*a*_*ijl*_*X*_*jl*_. *θ* denotes the sensitivity of dispersers to differences in fitness between habitats, and may be interpreted as the ability to assess habitat status. Larger *θ* represents higher certainty of habitat quality and *θ* = 0 indicates that no habitat information is available. Hence, when *θ* = 0 movements are random and when *θ* is increased the model approaches a step function of differences in habitat quality.

In each of the present iterated simulations, initial species abundances and parameters, *r*_*il*_ and *m*_*i*_, were randomly chosen from the uniform distribution U[0, 1], and *α*_*ijl*_ was randomly chosen from the uniform distribution U[0, 0.3]. These distribution ranges were chosen because system persistence becomes impossible when interaction strengths are large. Moreover, for simplicity, *e*_*ijl*_ and *s*_*il*_ were set to biologically feasible^48,49^ constant values of *e* = 0.2 and *s* = 1.0, respectively^38^. The habitat heterogeneity is expressed as randomly chosen parameters *r*_*il*_ and *α*_*ijl*_. All simulated habitats were connected to each other and simulations were performed with the same food web topology in all habitats.

Community persistence was calculated by measuring the frequency at which all species co-existed (Σ_*l*_*X*_*il*_ > 10^−13^ for all *i*) for periods that were sufficient (t = 10^4^) for community persistence to reach an asymptote in 500 runs per treatment. In each treatment, simulations were performed with randomly selected different combinations of parameters (*r*_*il*_, *m*_*i*_ and *α*_*ijl*_).

## Supplementary information

Supplemental figures

## Data Availability

All data generated or analysed during this study are included in this published article.
