# Supplementary material for: Adaptive migration promotes food web persistence
Source: Sci Rep. 2019 Sep 2;9:12632. doi: 10.1038/s41598-019-49143-8 (PMC6718410; doi:10.1038/s41598-019-49143-8)

Supplementary Information

Adaptive migration promotes food web persistence

A. Mougi

Supplementary Figure S1. Relationships between spatial coupling strengths (M) and stability in a random network model; (a) effects of adaptive dispersers (p_A) when $\theta = 50$; (b) effects of adaptive ability (θ) when $p_A = 1.0$. $N = 20$, $C = 0.5$ and $H_N = 2$.

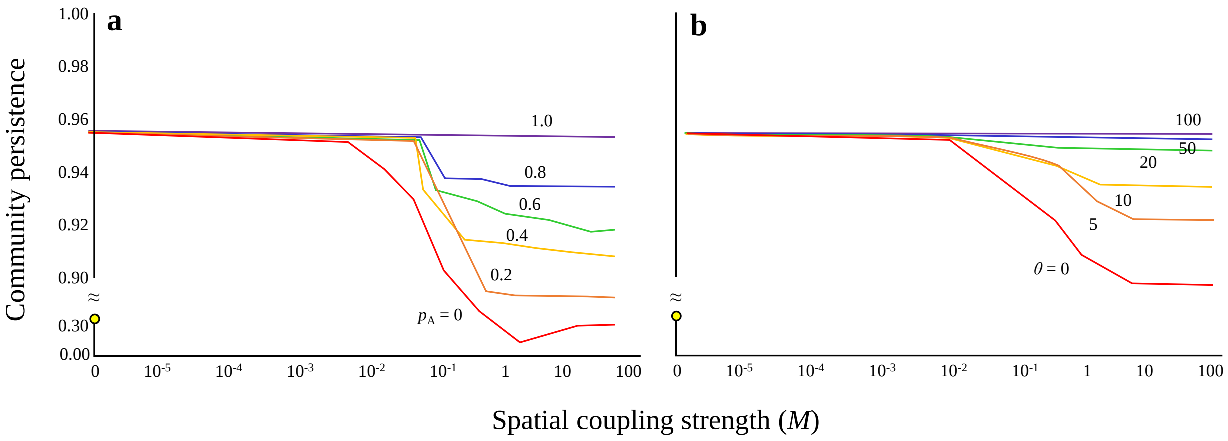

Supplementary Figure S2. Effects of adaptive ability on local extinction and global persistence; the proportion of local non-persistence is calculated as (1 – the number of times local populations of all species persist in all patches)/100 simulation runs. Higher non-persistence values indicate that local extinction is likely to occur, whereas lower values indicate a high chance of persistence across habitats. Colours represent different numbers of habitats. The cascade model is assumed; N = 20, C = 0.5, M = 1.0 and p_A = 1.0.

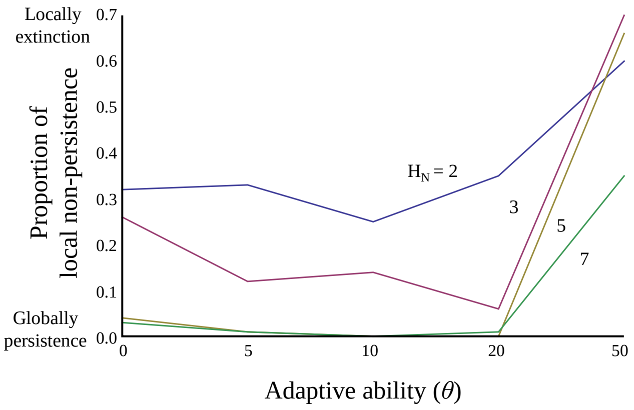

Supplementary Figure S3. Effects of adaptive abilities on dynamic behaviours of community systems; (a) mean abundance of all species; (b) mean minimum abundance of all species; a sufficiently long simulation run achieved an asymptotic state in which amplitudes (minimum densities) of population oscillations for all species did not change over time. Mean densities and mean minimum densities of all species across 100 simulation runs. Error bars indicate standard deviations. The cascade model is assumed; N = 20, C = 0.5, M =1.0 and p_A = 1.0.

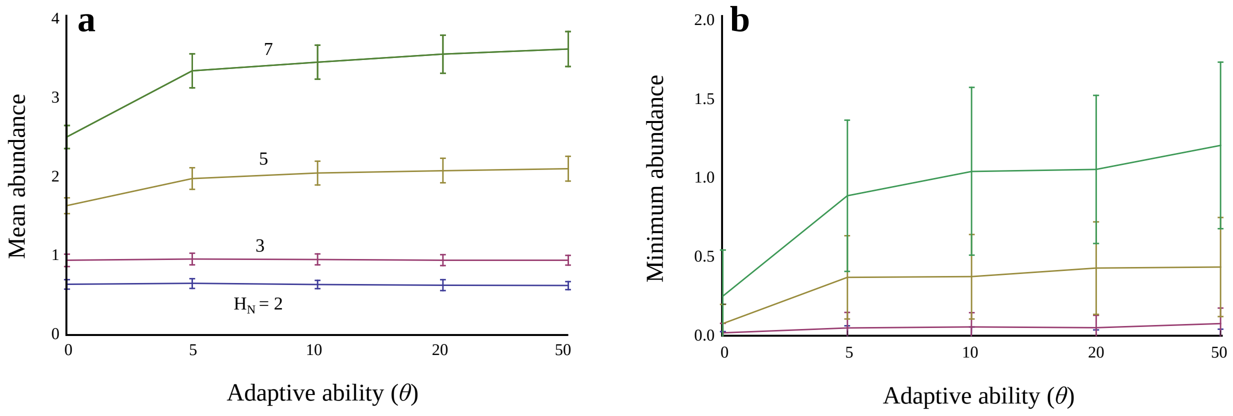

Supplement: Supplementary file 1 — Supplemental figures [file 41598_2019_49143_MOESM1_ESM.pdf]
